# Capturing the Formation and Regulation of Emotions in Collaborative Learning: The FRECL Coding Procedure

**DOI:** 10.3389/fpsyg.2022.846811

**Published:** 2022-04-25

**Authors:** Nikki G. Lobczowski

**Affiliations:** Learning, Research, and Development Center, University of Pittsburgh, Pittsburgh, PA, United States

**Keywords:** observational protocol, formation and regulation of emotions, collaborative learning, qualitative coding methods, learning analytics

## Abstract

Despite recent increases in research on emotions and regulation in collaborative learning, measuring both constructs remains challenging and often lacks structure. Researchers need a systematic method to measure both the formation of emotions and subsequent regulation in collaborative learning environments. Drawing from the Formation and Regulation of Emotions in Collaborative Learning (FRECL) model, I introduce a new observational coding procedure that provides comprehensive guidelines for coding these phenomena. The FRECL coding procedure has been implemented successfully in other studies and is described here in detail. Specifically, I detail the ideal situations for using the procedure, discuss background information and present a codebook and empirical examples for each stage of the FRECL model, and provide additional considerations that allow researchers flexibility based on their own experiences and preferences. This procedure extends past research by providing an accessible observational protocol that is both systematic and comprehensive. The FRECL coding procedure can benefit future research by providing more organized consistency to the measurement of collaborative emotions and regulation.

## Introduction

Research on collaborative learning has grown considerably in the past few decades (Dillenbourg et al., 1996). As researchers continued to try to understand small group learning, an imbalance of attention emerged in favor of cognitive processes (Baker et al., 2013). Recent shifts, however, have started to turn focus to other important aspects of learning, such as emotions. Despite these advances, researching emotions in collaborative learning remains a challenge due to the novelty of measuring emotions in small groups (Turner and Trucano, 2014). Current methods are often difficult to implement, inconsistent, incomplete, or inaccessible. Recent advances in theory of emotions in collaborative learning, however, have led to more optimal opportunities for measurement. The Formation and Regulation of Emotions in Collaborative Learning (FRECL; Lobczowski, 2020) model presents a comprehensive explanation of how emotions are both formed and regulated in small group learning. Existing research typically focuses on the formation of emotions (e.g., Pekrun et al., 2002) or socioemotional regulation (e.g., Näykki et al., 2014), but rarely both. More research is needed on how to capture emotions that manifest in collaborative groups and investigate the socioemotional processes to understand how and why student groups regulate their emotions. In this article, I will review existing measurement methods, describe a new coding procedure derived from the FRECL model, and discuss recommendations for subsequent data analyses while providing empirical support throughout.

### Measuring Emotional Constructs

Researchers can draw from the variety of methods used in different fields to conduct empirical research on socioemotional regulation. It is important not only to consider how to measure emotion regulation but also emotional responses, as they often signal to researchers that there are indeed emotions to regulate (Kappas, 2013). A comprehensive list of all types and examples of measurement for emotions is beyond the scope of this article, but there are some that are particularly salient for social emotions researchers.

#### Self-Reports

Many researchers measure emotions using self-report surveys (Pekrun et al., 2002; Russell and Barchard, 2002; van Kleef, 2016). Researchers often use these surveys to assess students’ perceptions of their emotions, which may not align with their actual emotional experiences. These measures are often “offline” in that researchers collect them after (e.g., minutes, hours) students experienced the emotions. They are often found to be less accurate than measuring emotions in the moment as they occur, as students may have difficulty remembering exactly how they were feeling or what caused the feelings (Kistner et al., 2010). Many scholars point out the drawbacks of self-reports in general, but they dominate the field nevertheless. This could partly be due to convenience and ease of administration.

Examples of the use of self-reports in the educational psychology literature on emotions or emotional regulation are abundant. In the educational psychology field, the Academic Emotion Questionnaire (AEQ; Pekrun et al., 2002) and Motivated Strategies for Learning Questionnaire (MSLQ; Pintrich, 2004) are quite prominent. Researchers use these to measure students’ emotions and regulation strategies, with the latter example also measuring motivational constructs. In the socioemotional literature, the most common self-report is the Adaptive Instrument for Regulation of Emotions (AIRE; Järvenoja et al., 2013). The purpose of this assessment is to pinpoint the socioemotional struggles that students experience in collaboration, as well as individual and group level efforts to regulate these challenges.

#### Think Alouds

Another popular measure for measuring self-regulated learning constructs, including emotion, is to have the students verbally express their thoughts as they complete a learning task (Greene et al., 2011). This concurrent think-aloud process is considered “online” and, if administered correctly, includes asking students to report their thinking rather than explaining it, which should not interfere with cognitive processes (Greene et al., 2011). Retrospective think alouds are conducted similarly but administered after the learning task (Azevedo et al., 2017). These are done by asking students to recall from memory or by showing them a video of themselves and asking them to recall what they were thinking. Either way, retrospective think alouds are “offline” and may not accurately capture emotional experiences.

#### Technological Tools

Researchers can use different technological tools to capture and subsequently measure emotions. Perhaps the most commonly employed is video and audio, but these are often used in conjunction with other tools. For example, researchers can also integrate eye tracking and facial coding systems (Azevedo et al., 2017). Eye tracking can capture patterns of eye movements and fixations on certain aspects of the learning environment, which can provide information about students’ interests or what they find relevant in a learning environment. Researchers can also use an automatized version of the Facial Action Coding System by Eckman and Friesen (1978), which uses patterns in facial muscles to determine emotions (Azevedo et al., 2016). For example, different combinations of movements of one’s eyebrows, mouth, and nose can indicate unique emotional expressions. This information can inform researchers, teachers, or pedagogical agents (i.e., computerized tutors) when students need assistance. Unfortunately, many facial coding softwares are difficult to use in collaborative settings due to their reliance on limiting the movement around the mouth. In some cases, tools can serve both intervention and measurement purposes. For example, Järvelä et al. (2015) suggested that tools for regulation should create awareness, promote sharing, and prompt regulation. On the back end, however, these tools provide a multitude of log data (i.e., often self-reports) that researchers can use to determine what students are feeling, the reasons for these feelings, and strategies they want to use to address these feelings (Miller and Hadwin, 2015).

#### Physiological Measures

Recently, researchers have explored different methodologies to measure emotions. For example, Pekrun et al. (2002) studied changes in cortisol to measure stress before and after an exam, finding that this physiological measure was more consistent at determining emotional intensity than self-reports. Other ways to detect arousal are through heart rate monitors (Järvelä et al., 2016a) and electrodermal activity through skin conductance responses (Azevedo et al., 2017). Researchers use these measures to not only determine arousal but also match it with other expressions of emotions (Azevedo et al., 2017) or sync them with similar data from other participants in similar social settings (Järvelä et al., 2016b). With these methodological advances, researchers now have more access to choose methods that best align with their contexts, research questions, and participants’ needs. However, physiological measures may not be available or desirable in given situations (e.g., distracting for younger children, secondary data analyses).

#### Observational Methods

Some researchers use observational methods to view students (i.e., in person or using video recordings) to determine the students’ emotional responses through their verbal and facial expressions as a substitute for self-report measures of feelings (Kappas, 2013; Azevedo et al., 2017). Observational measurements are often an accessible, less obtrusive method, and when video recordings are included, allow researchers the opportunity to spend sufficient time analyzing and interpreting the phenomena. Researchers can focus on what emotions students actually experience by interpreting their expressions as signs of emotions rather than relying on the emotions that they identify (van Kleef et al., 2016). Frijda (2016) suggested that researchers can infer emotions in others from their physiological reactions (e.g., sweating), behavior changes, or overreactions resulting from a new event. As social creatures, humans use their implicit knowledge about the context, participants, and behavioral indicators to infer what emotion the person experiences (Reisenzein et al., 2014). As such, researchers can interpret students’ expressions as signs of their emotions (van Kleef et al., 2016).

### FRECL Model Overview

In this paper, I will describe the Formation and Regulation of Emotions in Collaborative Learning (FRECL) coding procedure, which was derived from Lobczowski (2020) FRECL model. Similar to emotion models in psychology (e.g., Gross, 1998), the FRECL model included four stages of socioemotional formation, including *context*, *stimulus event*, *appraisal*, and *emotional response*, followed by a fifth stage for *regulation*. The *context* stage of the FRECL model emphasizes how interpersonal (i.e., person, population, and group) and task components interact to form specific factors that influence emotion formation and regulation. Interpersonal components include cognition (e.g., prior knowledge), motivation (e.g., value), emotions (e.g., anger), and behaviors (e.g., norms; Bakhtiar et al., 2018) across three foci: task (e.g., content knowledge), non-task (e.g., joking) and social (e.g., supporting others; Wolters, 2003). The *stimulus event* stage captures the task, non-task, and social factors that trigger emotional responses. Attention to the event is imperative at this stage (Gross, 1998, 2015), so it is possible that events may impact individuals within a group differently. Next, the students form *appraisals*, which are cognitive evaluations of the stimulus event against their motivational constructs. Specifically, the students judge (i.e., with or without awareness of doing so) how a stimulus event connects to their goals, values, and other motivational beliefs. This appraisal will then determine the students’ *emotional responses*. That is, an emotion will form, and the students may express it and experience physiological responses related to the specific emotion they experience. Finally, the students may or may not engage in the *regulation* stage, which includes a variety of strategies to control their emotions if they are aware of their emotions and choose to do so.

The FRECL model highlights both socioemotional formation and regulation, allowing for a better conceptualization of emotions in group learning. Importantly, the FRECL Model expands on traditional models for academic emotions by integrating social components within each stage (e.g., social appraisals). It also incorporates an interpersonal level (i.e., I, you, we) that considers how stimulus events can affect students within a group differently, which has implications for subsequent emotional development (e.g., shared or conflicting appraisals) and the mode of regulation (i.e., self-, co-, or socially shared- regulation of learning).

### Purpose

This paper describes an accessible option for measuring the formation and regulation of emotions in collaborative learning environments. The FRECL model observational coding procedure provides clear, comprehensive guidelines for capturing both the formation and regulation of emotions, which has been lacking in the existing literature. Specifically, in this paper, I will discuss how to code for the various stages of emotion formation and regulation, including complete codebooks and case examples, provide more information about when to use the procedure, and discuss additional considerations for researchers who require more flexibility.

## Considerations for Data Collection and Preparation

The FRECL coding procedure includes processes for measuring and understanding the formation and regulation of emotions in collaborative learning. This procedure was created to qualitatively capture the phenomenon of emotions in its entirety in order to understand both the causes and strategies used to regulate the emotions. In doing so, the procedure aids in the researchers’ abilities to understand the formation and regulation of emotions more in-depth. Conversely, it is also possible to quantify the deductive codes in the procedure to better understand the frequency or temporality of certain constructs. Additionally, the procedure can be applied to both short-term (e.g., one class activity) or longitudinal (e.g., groupwork over a semester) data to explore patterns in emergent emotions. Thus, the FRECL coding procedure provides a versatile method of measuring emotional constructs.

The FRECL coding procedure is most advantageous in authentic learning environments where students are working in small groups during a learning task. Although details for designing collaborative tasks are beyond the scope of this article, it is important to note that more complex tasks (e.g., project-based learning, more than one solution, requires discussion and argumentation) are more likely to produce rich data with observable emotional expressions (Meyer, 2014). The FRECL coding procedure is most ideal in those environments in which other measurements (e.g., heart rate, temperature, self-reports) are not optimal or available. That is not to say that these measurements are inadequate, but rather that they are not always preferable or accessible. Finally, given the depth of coding required, to optimally use the procedure, I suggest analyzing transcripts of video data (i.e., one camera per small group) rather than applying the observational protocol in real time. If possible, including timestamps on these transcripts can help the researchers analyze the temporality of different emotional constructs. Moreover, ensuring clear sound quality and views of students’ faces and movements will further enhance the coding process.

One last step before beginning to code is to consider the unit of analysis. With collaborative learning in general, and this work specifically, I suggest using a meaningful episode (Järvelä et al., 2016b; Järvenoja et al., 2017; Isohätälä et al., 2018), which consists of a collection of talk turns (i.e., episode) that become significant based upon the study’s research questions. For example, if the aim is to understand what events lead to group-level emotions, a meaningful episode could be a collection of student discourse in which a salient stimulus event theme occurred and triggered an observable emotional response. Another study may want to connect regulation strategies to specific emotional events. In that case, a meaningful episode could be passages in which an observable emotional response emerged and subsequent interactions to determine regulation behaviors that followed. Furthermore, many researchers do not code off-task discussions, but previous work has shown that emotional expressions can emerge in off-task discussions and may have important contextual implications for group productivity and functioning (Lobczowski, 2019; Lobczowski et al., 2021). Thus, I suggest including all student discourse when coding. There are many possible uses for the FRECL coding, but forethought about the data collection and preparation will optimize the implementation of the procedure.

## The FRECL Coding Procedure

In this section, I will discuss the successive steps of the FRECL coding procedure and provide a codebook and empirical examples for each. This protocol has been used in several empirical studies, but I will draw from one specific study to provide empirical support for the procedure (Lobczowski, 2019); for more information about the excerpts and conclusions drawn, please see the original study. In the exemplar study, my research team observed groups of pharmacy doctoral students during group meetings as they worked on a project-based task over a 6-week period, using an extreme case sampling with three groups that rated their feelings about collaboration as high, medium, and low over time. Specifically, we set out to explore patterns among the stimulus events that triggered emotions in collaborative learning. Thus, a meaningful episode was “a collection of student talk turns in which a salient stimulus event occurred and triggered an observable emotional expression at the individual, peer (i.e., a few students), or group level” (Lobczowski, 2019). The FRECL coding procedure was optimal for this context, given that there were too many differences between groups at each timepoint (e.g., project topic, assigned facilitator, previous interpersonal issues) to compare the groups quantitatively and that there were overarching longitudinal similarities (i.e., similar tasks to complete their project) that afforded a more qualitative exploration in thematic patterns. Moreover, the group’s meetings were loosely structured; groups would meet for up to 2 h (i.e., leaving at their own discretion) and choose their own discussion foci. Analyses of this type of “messy” environment were greatly benefited through the systematic nature of the FRECL coding procedure and would have been more challenging with other types of measurements.

This study also necessitated an important challenge of measuring emotional constructs using the FRECL method as a theoretical framework. The FRECL model shows a clear progression of emotion development backed by literature from various fields (e.g., Gross, 1998). Specifically, the emergence of an (observable emotional response) is the last stage in the formation part of the model. However, when coding for observable emotions, researchers must begin with this before coding for stimulus events and appraisals in order to capture the students’ responses to the events, rather than what response the coders expect. That is, coding stimulus events first would require researchers to predict the events that would generate emotional responses, thus integrating their own biases into the coding. Instead, the FRECL coding protocol recommends the following order for coding: (1) adding context, (2) emotional expressions, (3) stimulus events (i.e., those specific to the emotional expressions in step 2), (4) appraisals (again related to step 2). This may require researchers to engage in a more non-linear coding process, moving back and forth through an episode to capture the entire process, but it allows for a more descriptive, rather than prescriptive, account of the emotional events. Furthermore, upon first employment, a combination of deductive coding from the literature and inductive coding of emergent data was used. Similarly, researchers should not restrict themselves and allow the inclusion of their own emergent codes. It is important to continue to understand how emotions are formed and regulated in new contexts.

### Step 1: Adding Context

Before coding, it is important to add context to the transcripts, as detailed descriptions of events not caught in discourse can provide key information for data analysis (Rogat and Linnenbrink-Garcia, 2013). This is true for most qualitative research, and the types of details added to the contextual descriptions will depend on the research questions. Here, because we are focusing on emotion formation and regulation in collaborative learning, it is important to capture and note group interactions, group and individual behaviors, and background information from other sources (e.g., survey responses, previous group meetings) when possible. Using the context stage of the FRECL model as guidance, when coding for socioemotional formation and regulation, it is important to observe and record how the students interact during both task- and non-task-related discussions, as well as social interactions and behaviors between different students. This information can be vital in interpreting students’ verbal and non-verbal expressions.

Creating elaborated running records (Rogat and Linnenbrink-Garcia, 2011, 2013) before coding allows researchers to capture how interpersonal and task components impact the group members. Specifically, by adding additional forms of communication (e.g., head nodding, eye-rolling, pointing, laughing) to the transcripts, researchers can partner both verbal and non-verbal data for deeper analyses. This information could be added throughout coding but would likely be less reliable when trying to code for other constructs simultaneously. Conversely, analytic memos are designed to be added throughout the coding process to provide additional context and document emerging patterns and analytic ideas (Saldaña, 2016). As researchers pick up on patterns or gain additional insights into student interactions, temporal events can be noted, and richer interpretations can be made. It is important to note that adding context to data can be extra advantageous when transcripts are not available. If researchers can only code the videos directly, they can still add tags or notes (i.e., depending on the software used) that can be helpful in adding context that will augment data analyses.

#### Codebook

There is not a definitive codebook for context, as different settings, learners’ ages, and study durations would necessitate different considerations. Perhaps the most common are the non-verbal forms of communication mentioned previously. Researchers can also include the intended target or recipient of each students’ communication. For example, it will likely be important to note at whom someone is rolling their eyes or pointing. Drawing from the context stage of the FRECL model, researchers can also add important information related to students’ cognition (e.g., discrepancies in prior knowledge, task strategies), motivation (e.g., goals, relevance, values), emotions (e.g., emotional tendencies), and behaviors (e.g., norms, rituals), at both individual and group levels.

#### Empirical Example

In the low self-rated group from the model study, we noticed many negative interactions between two students, Liz and Don. Before coding, we added notes about their non-verbal forms of communication (e.g., when Liz rolled her eyes at Don) and important considerations for their utterances (e.g., sarcasm). Over the duration of the study, however, more and more information emerged about their turbulent past interactions. In later group meetings, when either Liz or Don was absent, they would discuss their feelings about the other, something they would unlikely do if they were present, which provided key contextual information for our data analyses. It was apparent from the videos and transcripts that their clashing personalities resulted in many disagreements. It was not until we coded a conversation that occurred after Liz and Don had left, though, that we heard their peers discussing an intense argument between the two in another class that we truly understood the importance of their previous interactions. The other group members were discussing how Liz and Don fought in another class. Through these additional conversations, we were able to conclude that Liz and Don’s negative interactions extended beyond the current context, as they brought negative perceptions of each other into the group from the outside exchanges.

In Excerpt 1, Liz brings up their mutual dislike for one another. Without context, Liz’s statement in Turn 5 could have been interpreted as a negative interaction that caused a tense situation, and Don’s response in Turn 6 would have been difficult to interpret. By adding more context to the episode (i.e., in italics below), however, it was clear that despite their negative opinions of each other, they were able to joke and laugh about their mutual dislike for each other.

**EXCERPT 1:** Maybe that’s why we don’t get along.

1	Liz:	So, this business stuff Don, do you just know it?
2	Don:	Just yep. I’m a little Warren Buffet. No, I’m not.
3	Liz:	I wouldn’t be surprised.
4	Don:	I’m not
5	Liz:	(*jokingly*) Maybe that’s why we don’t get along.
6	Don:	(*laughs, snorts, smiles*) Yeah, right?

### Step 2: Measuring Emotional Expressions

Next, it is important to capture the events that elicit emotional responses (Lobczowski, 2020). However, given that stimulus events are often harder to observe without emotional expressions (i.e., as students may react to them differently or not all), the FRECL coding procedure focuses first on capturing the emotional expressions. This allows for researchers to know for certain that a stimulus event was present (i.e., rather than hypothesizing what would trigger an emotional expression) and then analyze the text to determine the root cause.

Generally, measuring emotions in authentic settings is quite difficult. It is more attainable to code the emotional expressions shared through statements and non-verbal gesturing. Given the social nature of learning in collaborative environments, observable emotional expressions have important implications for group functioning; perceptions of emotions are just as important, if not more so than the emotions experienced by group members. For example, if a student suppresses their anger at another student, it will be difficult to capture it using an observational coding procedure, but the student’s anger will likely go unnoticed by the other group members, as well. Instead, the FRECL coding procedure is focused on capturing group-level emotional experiences; therefore, only the individual emotions shared in the group are captured. At the same time, research has shown that suppressed emotions could likely manifest in other ways (e.g., increased distraction, decreased responsiveness, lower peer rapport; Bonanno and Burton, 2013), in which case the FRECL coding procedure is situated to capture these as context for future interactions. This point supports another suggestion, in that it is important to capture non-speakers’ expressions (e.g., eye-rolling), as well. For this, researchers may need to add additional lines within their transcript and develop rules for distinguishing between verbal and non-verbal forms of communication (e.g., using italics for non-verbal).

To further strengthen the coding processes, I have created a five-level system for inference to reduce the potential for error. Using Reisenzein et al. (2014) foundations for observing emotions, the FRECL coding procedure focuses on specific phenomena (i.e., explicit and suggestive statements, facial expressions, body language, and tone), includes an observation protocol with clear definitions and examples (see “Codebook” in each section), and encourages the use of multiple coders to ensure reliability (see section “Additional Considerations”). By coding for explicit statements of emotions, suggestive statements of feelings about stimulus events, facial expressions, body language, and tone, researchers can strengthen the validity of their coding of emotional expressions. Moreover, these codes overlap and strengthen our context coding (Calvo and D’Mello, 2010) and can provide insights into appraisals (see Step 4 below).

#### Codebook

The FRECL model splits the emotional response stage into two distinct components: emotion and response. First, the emotion considers what the student(s) felt, including valence, activation, intensity, family/type. Given the difficulty of capturing these using observational methods alone, the FRECL coding procedure codes emotions by family/type, which are separated naturally by valence (Table 1) without trying to differentiate emotions by intensity or activation. For example, the procedure groups annoyance, frustration, and anger together, as the main difference between these emotions is the intensity of the negative feelings.

**TABLE 1:** Emotional expressions codes.

Code	Definition	Examples
Excitement/Enjoyment	Expressing enthusiasm in an activity	“We got a really good grade!”; smiling, excited tone, louder voice
Happiness/Joy	Expressing delight or contentment	“I’m glad that we got this done today;” smiling, content tone
Confidence	Expressing sureness of success, referring to past performance	“We did a really great job!”
Hopefulness/Optimism	Expressing hopefulness of success, referring to future performance	“I think we are going to do well.”
Relief	Being glad that something negative turned out positive	“I feel so much better now;” body relaxes
Boredom	Expressing signs of disengagement	“This is so boring”; appearing uninterested, detached from the task
Annoyance/Frustration/Anger	Expressing great dissatisfaction	“This sucks!” frowning, tense tone, louder voice
Disappointment/Sadness	Expressing mild dissatisfaction	“Our grade was lower than I expected,” frowning, lower voice
Dread/Worry/Anxiety	Expressing concern or nervousness about future events	“I’m concerned that we won’t get it done on time.”
Embarrassment/Shame	Judging failure as caused by oneself	“I’m so sorry that I let you down,” lower voice, poor posture
Hopelessness	A certainty of failure or undesirable outcomes	“It doesn’t matter what we do. We’re still going to fail.”
Shock/Surprise	A startled response due to an unforeseen event	“Wait, he said what!?!” louder voice, raised eyebrows
Stress	Expressing concern about past/present events	“We don’t have time for this!”

The response component focuses on how the emotion is conveyed, including expressions and physiological responses, which are the deliberate and involuntary reactions, respectively. Again, the FRECL coding procedure focuses on capturing the observable responses, which align with the five-level system of inference codes (Table 2).

**TABLE 2:** Levels of inference codes.

Code	Definition	Examples
Explicit statements	Students explicitly monitor their emotions	“This is so frustrating.”
Suggestive statements	Students express the cause of emotions or a statement that insinuates an emotion	“This class is the worst.” “This sucks.”
Facial expressions	The students use their faces to exhibit signs of emotions	A student furrows their eyebrows or rolls their eyes.
Body language	The students use their bodies to exhibit signs of emotions	Students throw their hands in the air.
Tone	Voice fluctuations in students’ voices exhibiting signs of emotions	A student raises their voice when confronting a peer.

#### Empirical Example

In the high self-rated group, Mary shared that she had previous research and work experience with a topic similar to the one for their class project, which was more unknown to the other group members. In the first two group meetings, Rick specifically called on Mary to help the group understand the content better by giving them all “a quick rundown of what are the transition points of care,” specifically “in the formal language that [Mary was] well-versed in.” After she did, he and other members of the group recognized the benefit of her expertise, as seen in Excerpt 2.

**EXCERPT 2:** Lucky to have Mary.

1	Brett:	Okay.
2	Rick:	We’re so lucky you’re in our group.
3	Mary:	(*laughs*)
4	Brett:	I know.
5	Mary:	Sorry, it’s just so, it’s so complicated. And I’ve. you’re right, I just like know it so well, so now I feel like I’m not projecting it well to you guys because I forgot that like, you guys don’t know, exactly.
6	Brett:	Yeah.

It was evident that the other students felt fortunate to have Mary in the group and were optimistic about their performance on the project. It was also clear, through Rick’s tone and body language (i.e., relaxed posture), that her explanation of the topic helped relieve some of his stress related to his lack of understanding of the content. In fact, Rick was likely engaging in emotional and cognitive regulation (i.e., seeking help) by asking Mary for “a quick rundown” of the topic to address both his content understanding and stress.

### Step 3: Identifying Stimulus Events

After the emotional expressions have been noted, the next step is to determine the root cause of the emotional expression. This will typically determine the beginning of the episode. For example, the episode above began when the group’s conversation shifted to the causes of their positive emotions (i.e., Mary’s expertise). Previous episodes during this group meeting were more focused on the negative emotions caused by the students’ confusion about the topic. Due to an abundance of possible root causes of emotional expressions, I recommend starting with common stimulus event categories that can later be explored, if necessary, during analysis. I also provide additional suggestions in later sections.

#### Codebook

The list of six codes in Table 3 was derived from a review of the literature on the most common causes of socioemotional interactions (Lobczowski, 2019). In my initial exploratory research, I left the events as categories to better capture patterns across the groups, then later explored the specific events through thematic analyses. For example, with the pharmacy students, external factors were a prevalent category that broke down into three main stimulus events: tests in other classes, grades, and work-life balance (Author). Given the unique setting of this past research (i.e., graduate students from a large cohort in a project-based learning environment), I would not expect other studies to necessarily have comparable stimulus events. Therefore, I suggest a similar approach when first analyzing unique contexts—begin with coding broadly and then explore later, if desired, to break apart into specific events.

**TABLE 3:** Stimulus events codes.

Code	Definition	Examples
Content and task	Understanding, planning, and strategies related to the content or task	Students disagree about the best strategy to complete the task.
Priorities	Priorities, expectations, and goals for the course, task, or collaboration	One student wants to get an A on the project; other students just want to get it done quickly
Communication	Verbal and non-verbal interactions between students	The students get frustrated when trying to negotiate a plan, as neither side is backing down
Working habits	Participation, commitment, focus, or quality standards	The group is angry at a student for not completing their part
Interpersonal dynamics	Personalities, interests, power dynamics, or past experiences	A group of friends enjoys working on a project together
External factors	Outside commitments, external events, or personal circumstances	Group members are frustrated with a student who misses meetings regularly for their job

#### Empirical Example

It was quickly evident that the medium self-rated group did not like the course or its central concept (i.e., innovation). As seen in Excerpt 3, the group had strong negative feelings about the course, including dread of completing the coursework and frustration and disgust at the mention of innovation, a topic that many of them did not intend to use in their future careers. Thus, the course, and here the word innovation, served as a stimulus even that triggered many negative emotions for several members of the group.

**EXCERPT 3:** I never want to hear that word again.

1	Beth:	Increase incentive for innovation.
2	Gabby:	[*mumbles*] by developing drugs.
3	Beth:	(*laughs*) Innovation.
4	Gabby:	Whoa (*as if to stop her from saying that word).* (*laughs*)
5	Anne:	I never want to hear that word again [.] Like when I hear it outside of pharmacy, I just like cringe.
6	Gabby:	To innovate, yeah, same. [*puking noise*]

### Step 4: Recognizing Appraisals

Appraisals are the evaluations of the stimulus events that students make (Gratch and Marsella, 2004), which include cognitive judgments about motivational constructs (e.g., causal attributions, control, value; Lobczowski, 2020). Appraisals are important for determining which type of emotion is formed. For example, if a group receives a low grade on their project (i.e., stimulus event), a student that is taking the class pass/fail due to low interest and relevance may exhibit a less-intense negative emotion than a student who blames the poor grade on the first student for not doing their part of the assignment, leading them to experience anger.

Notably, my previous coding experiences have shown that appraisals are often harder to capture, which is somewhat aligned with some theories on social emotions that state that appraisals are often automatic or subconscious and thus unlikely to be verbalized (Scherer, 2009). In the previous example about the low grade, a coder would need to be able to gather the information about the first student’s interest and relevance and then the second student’s causal attributions in order to accurately code for appraisals. Unfortunately, students may not express this due to social implications for doing so (e.g., mistrust due to an overreaction). Nevertheless, it is important to code for these when possible by focusing on statements about the stimulus event related to constructs such as goals, values, and other motivational beliefs. Moreover, appraisal codes may align with the codes in Table 2 (e.g., suggestive statements) and could receive additional support from adding contextual information.

#### Codebook

Using the FRECL model, appraisals are related to motivational constructs related to the stimulus event (Table 4). Statements (i.e., verbal or written) can help researchers understand the students’ perceptions of the stimulus events. Importantly, the appraisal stage is where the students’ reactions can start to differ and lead to disparate emotional responses, as seen in the previous example. Thus, it is imperative to capture appraisals whenever possible to fully understand the social situation.

**TABLE 4:** Appraisal codes—evaluations.

Code	Definition	Examples
Causal Attributions	Assigning credit to an event.	“It’s not my fault.”
Control	Assigning agency to an event.	“I couldn’t help it.”
Coping Potential	Determining the extent to which the event can be changed.	“It really doesn’t matter what we do. He will still fail us.”
Goals	Assessing how an event aligns with one’s goals.	“I’ll never get in the honor club now.”
Interest	Determining how the event connects to one’s interests.	“I don’t care about this at all.”
Relevance	Assessing how the event relates to one’s life.	“That has nothing to do with what we are working on.”
Urgency	Determining the timeliness of the event.	“We’ve got to finish. It’s due tomorrow!”
Utility	Assessing the usefulness of the event.	“I will never use this stuff.”
Value	Determining the importance or desirability of the event.	“I need to do well in this class to get a good internship.”

It is also important to capture when the appraisals converge, especially due to social intervention. Table 5 depicts codes for the interpersonal influences that lead to social appraisals (Manstead and Fischer, 2001; Lobczowski, 2020). These may be hard to code in singular episodes and may be more appropriate in analytic memos or during analyses by looking at similar stimulus events for a group and how the appraisals develop over time (e.g., one student sways others into similar appraisals).

**TABLE 5:** Appraisal codes—interpersonal influences.

Code	Definition	Examples
Emotional Contagion	Mimicry resulting from co-experience.	A student frowns when they see all the other group members frowning.
Social guidance	Letting others determine one’s reaction.	A student is not sure how to interpret a stimulus event and uses others’ evaluations to guide their own.
Social shared cognition	Group co-construction of an appraisal.	The group members create a shared evaluation of the stimulus event together.

#### Empirical Example

The medium self-rated group had a unique experience with their facilitator. She spent most of the time in their meetings discussing topics that the students thought were irrelevant to their task. This often led them to express frustration (e.g., negative tone, a furrowed brow, crossed arms) because she was wasting their time and not giving them much-needed guidance to understand their difficult topic. The group would spend time after she left complaining about her inadequacies, as seen in Excerpt 4, which highlights the evaluation of low relevance and low utility in the facilitator’s discussions.

**EXCERPT 4:** Not relevant to our future.

1	Anne:	[Fac] didn’t mention pharmacy.
2	Gabby:	I know. That’s why I was like, I don’t get… this doesn’t pertain to our future or what we’re currently doing.

Because the group thought the facilitator wasted time in their meetings, they felt more productive when she was not present. The group did not seem concerned that she had to miss several of their meetings and intended to capitalize on their extra time before she got back. Excerpt 5 highlights the groups’ low value in their facilitator’s attendance and support in general.

**EXCERPT 5:** It doesn’t matter that she is gone.

1	Anne:	And she’s not gonna be here for [a few sessions].
2	Gabby:	No. Not that it matters.

Across most of their group meetings, Gabby was the most talkative and animated and, in most situations, the loudest, suggesting that it was possible that her utterances may have led to social appraisals through social guidance. Considering that most of the group agreed and showed similar frustration throughout the semester, though, social shared cognition was also likely, especially in later meetings when the students felt more comfortable sharing their evaluations.

### Step 5: Capturing Regulation

Research on emotion regulation in collaborative learning environments has become more prominent in recent years (e.g., Bakhtiar et al., 2018; Mänty et al., 2020; Törmänen et al., 2021). When coding in conjunction with emotion formation, however, it is important to emphasize that students do not always engage in regulation after experiencing emotions (e.g., if they are unaware of their emotions; Gross, 2015). The FRECL model highlights how students may not be aware of their emotions or, even if they are, may choose not to regulate them (e.g., happiness; Lobczowski, 2020). It can be just as important, though, to capture and understand the episodes that lack regulation. Moreover, it is often doubtful to see individual regulation in group observations and is likely impossible when it is internalized (i.e., individual reframing).

#### Codebook

If the students engage in regulation, there are many processes for researchers to consider. First, they need to note the phase of regulation (Table 6). Similar to many models of self-regulation (e.g., Pintrich, 2000), the FRECL model suggests that students can engage in planning, monitoring/evaluating, controlling, or reflecting on emotions. Interestingly, the model expands previous theories, however, by further considering the types of strategy enactment in the control phase. Specifically, it states that students can plan a strategy to use in the future, engage in enacting a strategy, or reflect on a past strategy.

**TABLE 6:** Regulation codes—phase.

Code	Definition	Examples
Planning	Preparing to manage future expectations	Students anticipate negative emotions related to released test grades
Monitoring/evaluating	Discussing and/or expressing current emotions	“I’m so frustrated” or “Mark, I can tell that you are getting frustrated.”
Controlling – Planning Strategy	Planning to enact a regulation strategy	Students plan to take breaks during the meeting to manage future stress
Controlling – Enacting Strategy	Using a regulation strategy to change current emotions	“I’m getting frustrated. Why don’t we take a break?”
Controlling – Reflecting on Strategy	Evaluating past regulation strategies	Students discuss how taking a break helped them feel better and focus on the task
Reflecting	Judgments of past emotion control and adaptations for future	“I don’t think we handled our frustration well. Let’s take breaks at our next meeting.”

Next, given the social nature, it is important to capture the mode of regulation (Table 7), which captures who is engaging in the regulation. For this, regulation enactment can range from the individual- (i.e., SRL) to group- (i.e., SSRL) level. The regulation mode also includes CoRL, in which one or more individuals help other students in the group regulate their emotions. Importantly, the FRECL coding procedure distinguishes CoRL directed at another student or the whole group by coding the recipient as peer or group, respectively. Doing so allows researchers to capture individual behaviors and detect potential instances of a “more regulated other” (Lobczowski et al., 2021).

**TABLE 7:** Regulation codes—modes.

Code	Definition	Examples
Self-regulated learning (SRL)	Individual regulation of emotions	A student asks a teacher for help
Co-regulated Learning (CoRL)—Peer	One or more students help another student regulate their emotions	Two students sense a peer is frustrated and suggest a strategy that may help
CoRL—Group	One or more students help the rest of the group regulate their emotions	A student brings an activity for the group to help them improve their emotions after a hard test.
Socially Shared Regulation of Learning (SSRL)	Group-level regulation of emotions	A group co-constructs a plan to regulate their frustration caused by a bad grade

Finally, researchers can capture the regulation strategies that students use to regulate their emotions (Table 8). Due to the novelty of this research, I first inductively coded for strategies as they emerged. In a recent study, I compiled a list of the strategies used by the pharmacy students (Lobczowski et al., 2021). I found that it was easier to classify strategies within categories that varied by domain (i.e., cognitive, motivational, behavioral, and interpersonal). Table 8 depicts the codes from that study, as well as specific examples within each category. Within each category, connections to different stages of the model can also be seen (e.g., reframing addresses the appraisal stage).

**TABLE 8:** Regulation codes—strategies.

Code	Definition	Examples
Addressing understanding (cognitive)	Focusing on learning content to address misunderstanding.	Correcting misunderstandings; Finding information; Providing clarification
Adopting a new tactic (cognitive)	Trying something new when stuck on a problem.	Avoiding; Changing topic; Giving suggestions; Trying a different strategy
Disengagement (behavioral)	Avoiding the cause of emotions.	Being by yourself; Giving space; Giving up; Going for a walk
Looking ahead (cognitive and motivational)	Focusing on future events rather than current emotions.	Delegating to future self; Focusing on the future; Continuing moving forward; Making a plan; Planning to celebrate
Reframing (cognitive)	Change perspective of the appraisal of the stimulus event.	It could be worse; Normalizing; Perspective building; Rationalizing; Reappraising
Restructuring task (motivational)	Changing the task to improve task-related emotions.	Breaking down the problem; Prioritizing; Procrastinating; Setting manageable expectations
Showing empathy (interpersonal)	Acknowledging others’ emotions and/or trying to help address them.	Comforting; Encouraging others; Giving a hug; Offering to help; Pointing out strengths; Providing reassurance; Providing relief; Sensing distress; Showing concern; Validating others
Seeking help (interpersonal)	Asking others for help when needed.	Asking for help; Seeking validation; Talking to loved ones; Using a buffer
Using humor (interpersonal)	Integrating humor into conversations to improve emotions.	Joking; Making fun of oneself; Telling a funny story
Venting/complaining (interpersonal)	Discussing emotions (and their causes) with others.	Commiserating; Complaining about another group member; Sharing frustrations

#### Empirical Example

In the high self-rated group, Brett often provided comfort whenever other group members seemed to be struggling with their emotions. There were several instances in which Linda and Mary were upset over grades, tests, or graduate school in general, and Brett was there to give them a hug or say something nice. He often seemed to sense what others were feeling and would address it appropriately. In Excerpt 6, Rick had been pushing Mary to ask her mentor for a favor to help the group with the project, but Mary seemed apprehensive about contacting her mentor, who had just started her maternity leave. Brett, on the other hand, sensed her trepidation and backed off, which prompted Mary to open up and share the cause of her concerns, allowing the group to reach a compromise.

**EXCERPT 6:** We don’t want you to do anything that makes you feel uncomfortable.

1	Brett:	But I can tell we’re putting you in a very uncomfortable position, and I don’t like that.
2	Mary:	No, I just, I just don’t know like she’s gonna read it. You know what I mean? I don’t want to cross any boundaries with. Like, I don’t if she’s gonna think this is. Like I wouldn’t.
3	Brett:	Are you trying to find help sort of thing, or-
4	Mary:	A little bit of that, and like but honestly like I wouldn’t mind help, because like I’m the only student whose supposed to have like a fellow with me and they didn’t. But like, I guess it’s more. I honestly don’t know how to explain it.
5	Brett:	Well, think about it, because I, we don’t want to do something that puts you, that makes you uncomfortable.
6	Linda:	Yeah.
7	Brett:	So, if you can, like, need a minute to think about what you’re thinking, then.
8	Mary:	No, but I like drafting an email with you guys to see if it sounds, you know what I mean, to see if it sounds okay.
9	Brett:	But are you comfortable with even sending an email to her?
10	Mary:	Yes.

In this CoRL-Peer episode, Brett is monitoring and controlling Mary’s distress (i.e., worry) by engaging in several *showing empathy* strategies, including showing concern (i.e., Turns 5 and 9) and providing relief (i.e., Turn 7). Interestingly, although he is speaking to Mary and helping her regulate her negative emotions, he is also addressing the group to let them know it is not okay to put her in a compromising position with her mentor. This led to Mary engaging in *adopting a new tactic*, in which the group would help her carefully think through and construct an email with which she felt comfortable.

## Additional Considerations

The previous sections have provided details and examples about how to use the FRECL coding procedure. Although the procedure is systematic and comprehensive, there is room for some flexibility based on the researchers’ skill sets, preferences, tools, and accessibility. Thus, there are additional options to bolster and supplement the FRECL coding procedure.

### Reliability

With such a complex coding procedure, it is important to consider how to increase and accurately measure reliability. First, an easy way to increase reliability is to search for meaningful words and phrases. By this, I mean using different features of the chosen software (e.g., Control + F) to locate phrases that will likely be coded. For example, researchers can search for words or stems related to explicit expressions of emotions, such as “frustrated,” “frustrating,” or “frustrat*.” Doing so will help guarantee that instances of explicit statements are not missed during coding, which could be especially advantageous with large datasets. Importantly, I am not suggesting that these are automatically coded without consideration but rather as a means to improve reliability. Similarly, coders can search for other common suggestive statements, such as “this sucks” or common curse words. As they go through the transcripts, it is also likely that they will develop an understanding of the students’ verbal inclinations and can add to their list of common phrases for which to search. It is important to note that I do not suggest using this strategy in isolation but rather as a supplemental strategy to capture common phrases, which can be especially helpful with large datasets.

Next, consideration needs to be given to interrater reliability. As previously mentioned, to increase confidence in coding, using two coders increases the quality of the coding (Saldaña, 2016). With such a multifaceted codebook, however, that can be difficult. First, engaging in a calibration phase is critical (Saldaña, 2016). That is, researchers need to practice coding and work through understandings and interpretations with supplemental data before engaging with the data used for the analyses. The calibration phase also helps capture more inductive codes upfront to be added to the codebook before official coding begins, which in turn, can help increase reliability. Moreover, Grain size is important but can also impact inter-rater reconciliation efforts. This is a particularly important consideration given the multitude of codes. Whereas simple agreement across all codes allows researchers to inter-rate and reconcile across codes for each episode, the reliability measure will likely be misrepresented given that codes with high reliability might could those with lower reliability (McAlister et al., 2017). Inter-rating across each code type (e.g., regulation phase, mode, and strategy) allows for more accurate measures of reliability but can be extremely tedious, as researchers will need to spend more time documenting the discrepancies by each code type. Some softwares (e.g., NVivo), however, do have built-in solutions for measuring inter-rater reliability. Inter-rating becomes even more difficult with emergent coding. If an emergent code or important phrase occurs, it is important to go back and update all previously-coded episodes. Regardless, the key is to be systematic and thoughtful in making decisions (Reisenzein et al., 2014) and ensuring that all codes are reconciled prior to analyses.

### Learning Analytics Support

Learning analytics can also be used to add rigor and reliability to the coding and can be particularly appealing with large datasets. A full explanation of different learning analytical procedures is beyond the scope of this paper; instead, I will discuss a few that can supplement the FRECL coding procedure and will provide resources with more logistical information. First, similar to searching for common phrases, researchers can use machine learning to automatically code for explicit statements of emotions (e.g., “this is frustrating”; Sherin et al., 2018). Again, I would suggest that coders double-check each of these for accuracy.

Next, researchers can use different text-mining approaches to retrieve important information from the students’ utterances (Silge and Robinson, 2017). Another particularly pertinent method is sentiment analysis, which uses existing lexicons (e.g., AFINN; Nielsen, 2011) to assign valence or weighted scores to words from the groups’ conversations. Although these dictionaries could likely miss some words or ignore context, they provide a systematic way to capture valence across different students and groups. This information can help researchers determine case groups or meetings to study or can provide information on individual differences within groups. Similarly, topic modeling is a process that organizes text in a meaningful way. This method clusters discussion topics and presents an overview of the topics that can be applied to both small (e.g., episodes) or large (e.g., 2-h meetings) datasets. Importantly, it can also be applied to only those episodes coded or across all utterances. Researchers can use the overview of the topics discussed to support their coding and analyses or guide their coding from the onset. For example, topic modeling could be used to determine emergent stimulus events, which can be used during coding, rather than the ones suggested above that were derived from existing literature. Finally, data visualizations such as word clouds can provide clear pictures for comparing groups, students, or timepoints. These visual supports can help strengthen analyses or help researchers detect patterns during coding. Importantly, for each of these methods, researchers will likely want to use original transcripts (i.e., before adding context) or add in the added context as stop words to avoid altering the text-mining results.

### Recommendations for Data Analyses

Once researchers complete their coding, they will then use the resulting codes to draw conclusions from their data. Although each type of code provides key information about group processes, the interactions of each are also important to consider in order to understand the specific contexts. For example, when analyzing regulation, it may be important for researchers not only to identify the strategies (e.g., taking deep breaths) used but connect them to specific emotions (e.g., anxiety) or appraisals (e.g., low control, high relevance). The specific type of analysis should depend upon the research questions. In my previous work, I used thematic analyses to understand the common stimulus events within and across the three groups of pharmacy students (Lobczowski, 2019). Conversely, researchers could quantify the codes to look for relationships between the codes or connect the codes to other data (e.g., performance measures). Again, learning analytics can provide both guidance for analyses or support when reporting results. Data visualizations and topic modeling of stimulus events can provide a look into common issues faced across groups or can be used as a validity measure to check against other conclusions. In sum, the FRECL coding procedure provides a way to capture emotion formation and regulation in collaborative learning environments, which can support a variety of analyses to answer important research questions.

## Discussion

Measuring and analyzing emotions in collaborative learning is a fairly new field, and future researchers likely need support to do so systematically. Thus, I have presented the FRECL coding procedure, a clear and comprehensive protocol for capturing both emotion formation and regulation in collaborative learning environments. This procedure provides much-needed guidance for analyzing large, complex datasets and supports deep analyses and a more complete understanding of academic emotions than has been seen in recent studies. Moreover, the FRECL coding procedure allows for temporal connections between emotional components, a better understanding of specific situations, and the detection of nuances that highlight important contextual information. The FRECL coding procedure can be used in multiple contexts to answer a variety of research questions related to academic emotions. To allow more flexibility, additional considerations can provide alternatives to adapt to fit researchers’ skillsets, tools, and preferences.

### Significance

This new coding method provides a comprehensive way to measure the formation and regulation of emotions in authentic, collaborative settings. It also captures both individual and group processes, which is important to understanding socioemotional interactions. This protocol provides an alternative to existing measures that may be offline or obtrusive methods while also capturing the formation of emotions, which has often been neglected in previous research.

### Limitations and Future Directions

Despite efforts to combat limitations with typical observational protocols for emotions (i.e., the five-level system of inference), I acknowledge the probable inability to accurately capture every emotion experienced by students during collaborative learning. Nevertheless, the comprehensiveness of the FRECL coding procedure and its ties to the FRECL model make it a desirable solution in comparison to when alternatives are less accessible (e.g., insufficient funding) or appropriate (e.g., difficult classroom layout). On the other hand, the future of measuring emotions will likely be multimodal (Järvelä et al., 2019), in which multiple measurement methods are combined for improved accuracy and interpretation. For example, combining heart rate measures with the FRECL coding procedure may provide a way to measure intensity and help researchers better distinguish within different families of emotions. This highlights the importance of including timestamps in transcripts to link the different measures and better capture temporality. Moreover, by combining methods, researchers can also increase the likelihood of capturing individual emotions, assuming that is part of their research aims. My suggestion is for researchers to consider the environment, research questions, and tools necessary and available for analyzing collaborative emotions and make the best choice for their situation.

## Data Availability Statement

The data analyzed in this study is subject to the following licenses/restrictions: The data contains detailed conversations that may lead to the identification of the participants. Requests to access these datasets should be directed to NL, nikkilob12@gmail.com.

## Ethics Statement

The studies involving human participants were reviewed and approved by University of North Carolina Office of Human Research Ethics. The patients/participants provided their written informed consent to participate in this study. Written informed consent was obtained from the individual(s) for the publication of any potentially identifiable images or data included in this article.

## Author Contributions

The author confirms being the sole contributor of this work and has approved it for publication.

## Author Disclaimer

Any opinions, findings, and conclusions or recommendations expressed in this material are those of the authors and do not necessarily reflect the views of the EII.

## Conflict of Interest

The author declares that the research was conducted in the absence of any commercial or financial relationships that could be construed as a potential conflict of interest.

## Publisher’s Note

All claims expressed in this article are solely those of the authors and do not necessarily represent those of their affiliated organizations, or those of the publisher, the editors and the reviewers. Any product that may be evaluated in this article, or claim that may be made by its manufacturer, is not guaranteed or endorsed by the publisher.
